# Sequentially Vapor-Grown Hybrid Perovskite for Planar Heterojunction Solar Cells

**DOI:** 10.1186/s11671-017-2401-5

**Published:** 2018-01-11

**Authors:** Won-Gyu Choi, Dong-Won Kang, Sungjae Na, Chan-Gyu Park, Fatma Pinar Gokdemir, Taeho Moon

**Affiliations:** 1 Department of Materials Science and Engineering, Dankook University, Cheonan, 31116 South Korea; 2 Department of Solar and Energy Engineering, Cheongju University, Cheongju, 28503 South Korea; 3 Department of Physics, Yildiz Technical University, 34210 Istanbul, Turkey

**Keywords:** CH_3_NH_3_PbI_3-x_Cl_x_, Sequential vapor processing, Vacuum evaporation, Vapor-assisted growth, Planar Heterojunction solar cells

## Abstract

High-quality and reproducible perovskite layer fabrication routes are essential for the implementation of efficient planar solar cells. Here, we introduce a sequential vapor-processing route based on physical vacuum evaporation of a PbCl_2_ layer followed by chemical reaction with methyl-ammonium iodide vapor. The demonstrated vapor-grown perovskite layers show compact, pinhole-free, and uniform microstructure with the average grain size of ~ 320 nm. Planar heterojunction perovskite solar cells are fabricated using TiO_2_ and spiro-OMeTAD charge transporting layers in regular *n*-*i*-*p* form. The devices exhibit the best efficiency of 11.5% with small deviation indicating the high uniformity and reproducibility of the perovskite layers formed by this route.

**Electronic supplementary material:**

The online version of this article (10.1186/s11671-017-2401-5) contains supplementary material, which is available to authorized users.

## Background

Hybrid perovskite materials are the most competitive candidates as light absorber of next-generation photovoltaic era with their unique features including intense optical absorption, direct and tunable band gap, high carrier mobility, long charge diffusion length, shallow defect levels with few mid-gap states, and wide tunability on its composition according to metal halide framework and inserted organic species [1–8]. They have been employed in two types of architectures such as mesoscopic nanostructured and simple planar structured. The preparation of high-quality pinhole-free perovskite layers for simplified planar architecture requires substantial effort. Various methods have been used to prepare perovskite layers, such as anti-solvent dripping, sequential dip coating, dual-source vacuum evaporation, and vapor-assisted growth [9–16]. Vacuum deposition presents highly uniform layer formation over the entire substrate area, with thickness control ability. Furthermore, vapor-assisted crystallization is known to reproducibly provide densely packed microstructure through controlled chemical reaction speed via diffusion of organic material [17–26].

Here, we report a novel sequential vapor-processing route by CH_3_NH_3_I (MAI)-vapor diffusion into vacuum-deposited PbCl_2_ layers, resulting in fully covered and highly uniform perovskite layers. Planar *n*-*i*-*p* heterojunction perovskite solar cells are successfully demonstrated by employing TiO_2_ and 2,2′,7,7′-tetrakis-(n,n-di-4-methoxyphenylamino)-9,9′-spirobifluorene (spiro-OMeTAD) charge transporting layers. Champion cells achieve power conversion efficiencies (PCE) up to 11.5%. Our results show that this route is feasible to fabricate uniform and reproducible perovskite layers in a controlled way.

## Methods/Experimental Procedure

### Device Fabrication

Devices were fabricated on fluorine-doped tin oxide (FTO)-coated glass substrates. The substrates were sequentially cleaned in an ultrasonic bath by acetone, methanol, isopropanol, and deionized water and then exposed to ultraviolet-ozone for 15 min. For electron transporting layers, 450 and 600 mM titanium diisopropoxidebis(acetylacetonate) in n-butanol (75 wt% in isopropanol) were double-coated at 2500 rpm for 20 s and annealed at 500 °C for 30 min in air to form compact TiO_2_ layers. The TiO_2_-coated substrates were placed in a vacuum chamber, and PbCl_2_ was evaporated to a rate of 1 Å/s for ~ 16 min at room temperature. Methyl-ammonium iodide (MAI) vapor treatments were carried out in a drying vacuum oven using MAI powder spread around the PbCl_2_-coated substrates. Subsequently, the as-prepared black samples were washed with isopropanol for the removal of the MAI residue and then annealed at 100 °C for 1 h. For hole transport layers, precursor solutions were prepared by mixing spiro-OMeTAD in chlorobenzene with tert-butylpyridine and lithium bis(trifluoromethylsyfonyl)imide salt in acetonitrile. The solutions were spin-coated at 4000 rpm for 40 s, and then the coated samples were kept in air overnight for oxidation. Finally, device fabrication was completed by thermal evaporation of Au electrodes.

### Characterization

The crystal structure was analyzed by X-ray diffraction (XRD, Ultima IV: RIGAKU), and the morphology of the perovskite layer was observed by a field emission scanning electron microscopy (FE-SEM, S-4300: HITACHI). The optical absorbance data were obtained using a UV-Vis spectrophotometer (UV-1601PC: Shimadzu). The photocurrent density-voltage (*J*-*V*) curves of the perovskite solar cell devices were recorded with a solar simulator (94021A: Newport) under AM 1.5G (100 mW/cm^2^) irradiation. During the measurements, the solar cell devices were masked with an aperture area of 0.09 cm^2^.

## Results and Discussion

**Fig. 1:**
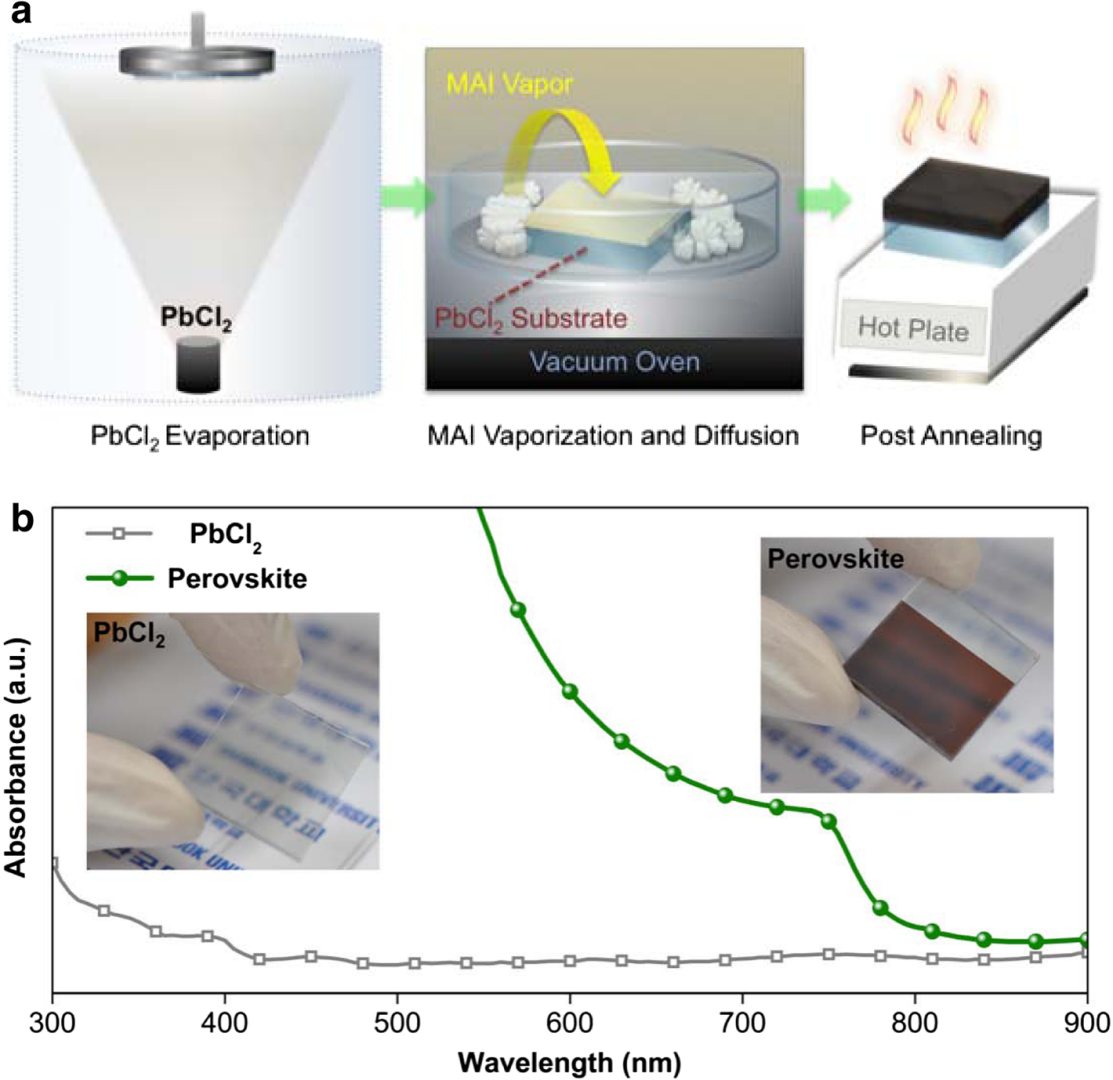
**a** Schematic illustration of the fabrication process, via PbCl_2_ evaporation, MAI vaporization and diffusion, and post-annealing. **b** UV-Vis absorption spectra of the PbCl_2_ and perovskite layers. Corresponding sample photographs are given in inset

A novel route using sequential vapor processing offers reproducible formation of pinhole-free, densely packed crystalline perovskite layers. Figure 1a briefly illustrates the fabrication process of the high-quality perovskite layers. Firstly, PbCl_2_ is evaporated in a vacuum chamber using an effusion cell on TiO_2_/FTO/glass substrates, producing homogeneous layers reproducibly over entire substrate area. Furthermore, well-defined deposition rate through vacuum deposition makes the thickness control of PbCl_2_ and resultant perovskite layers easy, compared to liquid processing. The obtained homogeneous and transparent PbCl_2_ samples are transferred to glass petri dishes while the coated sides are facing upward. For MAI vaporization, MAI powder spreads out around the PbCl_2_-coated substrates, and each petri dish is tightly covered with another glass lid on the top ensuring a well-confined space. In a vacuum oven, various temperatures and periods are monitored to find out the best condition for perovskite formation. Since the process is featured in MAI diffusion and reaction as well as its vaporization, a moderate condition is favored for reproducible formation of high crystallinity perovskite. Finally, post-annealing is performed to improve the crystallinity through the sufficient reaction of unreacted components. Figure 1b shows the optical absorption spectra of the PbCl_2_ and perovskite samples with the representative sample photographs. The dark-brown-colored homogeneous layer with the expected absorption edge around 785 nm indicates the successful crystallization of the perovskite by this route. Additionally, the bandgap value estimated from the Tauc plot (Additional file 1: Figure S1) is found to be around 1.58 eV that is in a good agreement with the literature [27, 28].

**Fig. 2:**
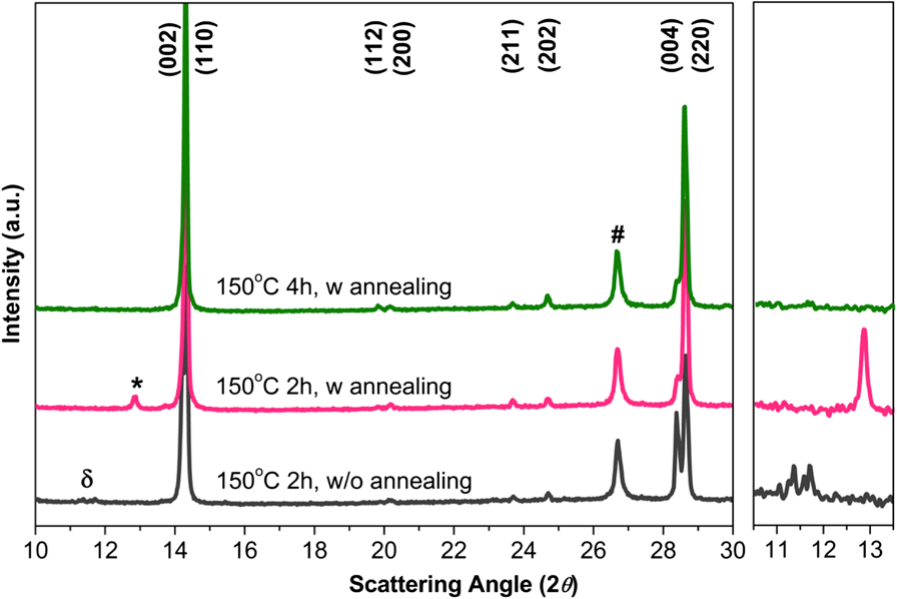
XRD data of the perovskite thin films, according to MAI processing time and post-annealing execution. The perovskite plane indices are assigned, and the peaks for the perovskite complex, PbI_2_, and FTO are also denoted as δ, *, and #, respectively

First, the effect of MAI process temperature on perovskite formation was roughly investigated using absorption spectra. As shown in Additional file 1: Figure S2, 150 °C was the optimum condition with the clear perovskite absorption edge, which is probably due to the optimization between MAI vaporization and perovskite formation reaction. Afterwards, more detailed investigation such as MAI process period and post-annealing execution was conducted, and XRD analyses were carried out to understand the evolution of crystal growth (Fig. 2). All the samples exhibit the characteristic perovskite diffraction peaks attributed to tetragonal crystal structure, and relatively strong intensities to the [001] and [110] directions verify that highly aligned crystal orientation was obtained [29–31]. Although the diffraction intensities of the second phases are very small, the phase transformation order can be grasped from their tendency according to the applied thermal energy. When MAI vaporization period is kept only for 2 h without annealing, some peaks appear between 11° and 12°. The previous studies reported these peaks were related to the H_2_O-incorporated perovskite complex ((CH_3_NH_3_)_4_PbI_6_·2H_2_O) that can be formed due to moisture and excess MAI [32–42]. With applying the post-annealing treatment (at 100 °C for 1 h), these peaks give place to a PbI_2_ peak, due to the release of moisture and/or MAI [32, 43, 44]. When increasing MAI vaporization period to 4 h with the post-annealing step, full conversion from PbCl_2_ to perovskite is obtained.

**Fig. 3:**
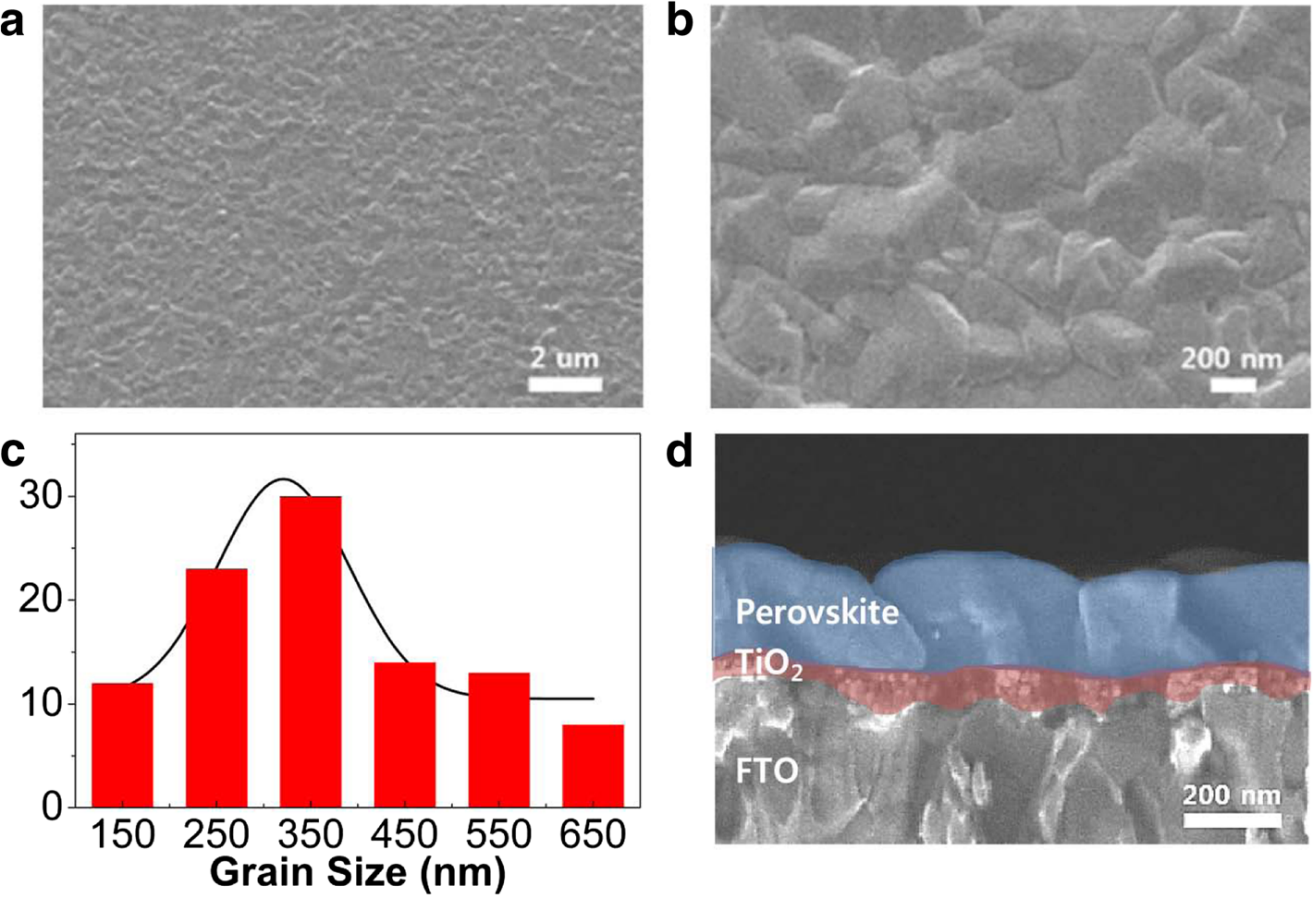
SEM analyses of the 220-nm-thick perovskite layer. **a** Low magnification image. **b** High magnification image. **c** Histogram showing the grain size distribution. **d** Cross-sectional view

Fabricating pinhole-free perovskite is essential for efficient planar solar cells. Our route utilizing physical vacuum deposition produces compact and uniform perovskite over full substrate area reproducibly. The investigation on the morphology and microstructure of the perovskite layers was performed by SEM analyses. Pinhole-free, uniform, and homogeneous surface characteristics are revealed with the low magnification SEM image (Fig. 3a). The closely packed grains with complete coverage are also seen in the high magnification mode (Fig. 3b). The mean grain size was extracted as ~ 320 nm using a Gaussian fit of the histogram as given in Fig. 3c. The cross-sectional view in Fig. 3d clearly reflects the distinct and continuously grown morphology of the perovskite layer. Moreover, the average perovskite thickness (~ 220 nm) is smaller than the mean grain size, ensuring the vertical transport of charge carriers through the grains.

**Fig. 4:**
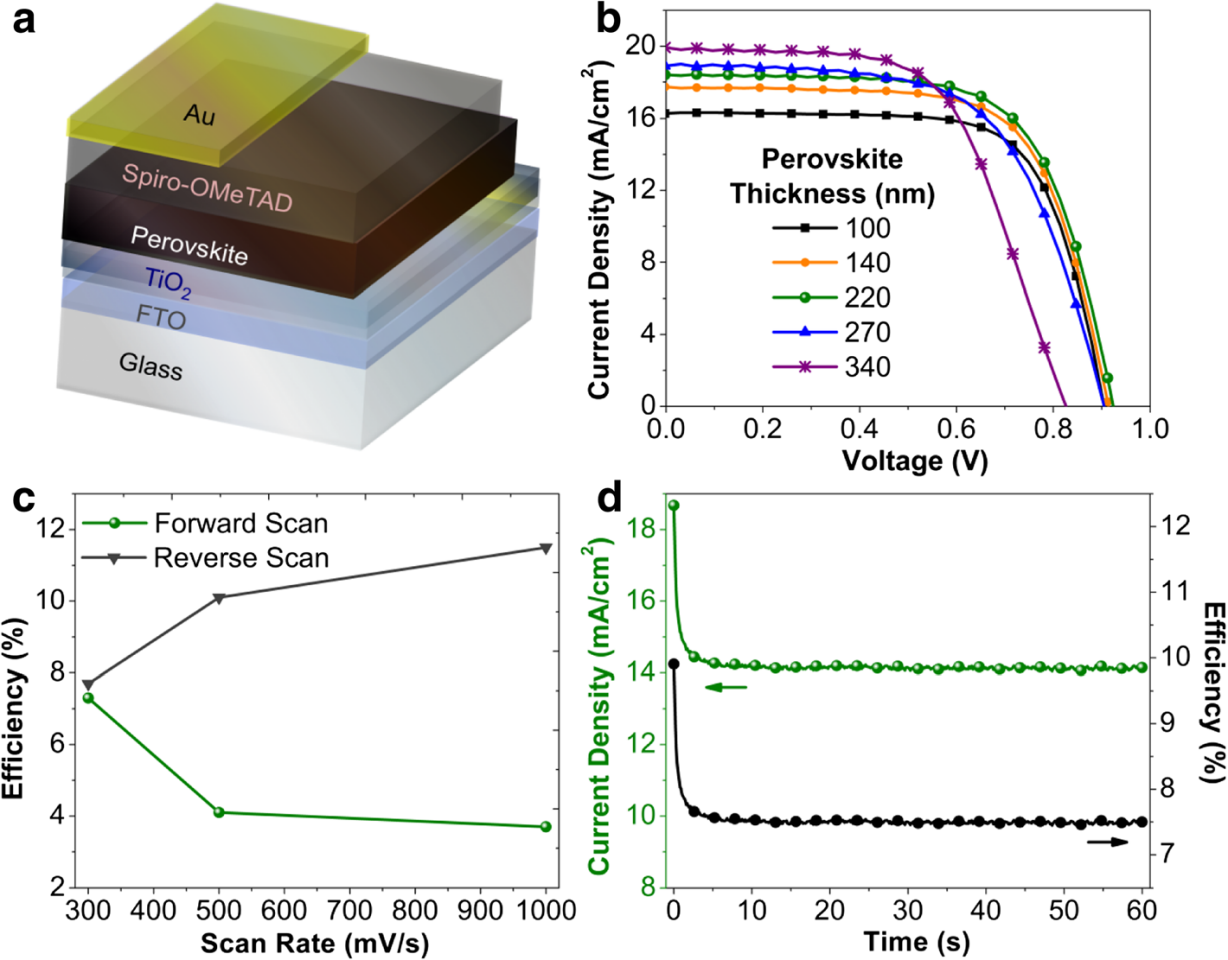
**a** Device scheme. **b** *J*-*V* curves of the perovskite solar cells with various perovskite thicknesses. 1000 mV/s with reverse scan. **c** Scan rate-dependent hysteresis change, perovskite thickness: 220 nm. **d** Stabilized output at the maximum power point voltage

The device scheme of the fabricated *n-i-p*-type planar heterojunction perovskite solar cells is given together with the representative *J-V* curves for five different perovskite thicknesses in Fig. 4a, b. The photovoltaic parameters extracted from the *J-V* curves are summarized in Table 1. It is worth noting that our vapor processing allows free thickness control through well-defined deposition rate thereby ensuring easy optimization of device efficiency. The optimized cells were found to have an average efficiency of 11.2% with the 220 nm perovskite thickness. The smaller optimum thickness, compared to that in the literature showing high efficiency through a solution process, points out that the charge collection ability of our perovskite should be further improved. It is necessary to develop perovskite layer quality with the vapor-processing route in order to mitigate carrier recombination. The standard deviation was calculated from three devices that made on the identical substrate for each condition. Despite the limited sample numbers, the small deviations indicate the excellent uniformity of the perovskite layers on the whole substrate area with this sequential vapor process. Figure 4c presents the hysteresis analyses as a function of scan rate for the 220-nm-thick perovskite device. The scan rate dependence on the hysteresis is clearly shown. At the low scan rate (300 mV/s), as given in Additional file 1: Figure S3, the hysteresis becomes negligible with the average efficiency of 7.5%. For *n*-*i*-*p* structure, hysteresis showing higher PCE at reverse scan is usual, indicating that carrier collection (i.e., transport and/or transfer at interfaces) is more efficient with a specific distribution of capacitive charge such as space charge and trapped charge. On the other hand, steady-state PCE was monitored at the maximum power point as given in Fig. 4d. The stabilized PCE and current density values were obtained as 7.5% and 14 mA/cm^2^, respectively, which is well matched to the result in Fig. 4c.

**Table 1:** Photovoltaic parameters of the perovskite solar cells with various perovskite thicknesses

Thickness (nm)	*J*_sc_ (mA/cm^2^)	*V*_oc_ (V)	FF	PCE (%)
100	16.30 ± 0.08	0.89 ± 0.02	0.72 ± 0.01	10.40 ± 0.02
140	17.57 ± 0.12	0.91 ± 0.01	0.67 ± 0.02	10.68 ± 0.39
220	18.33 ± 0.09	0.91 ± 0.01	0.67 ± 0.02	11.24 ± 0.34
270	18.73 ± 0.24	0.89 ± 0.02	0.61 ± 0.02	10.23 ± 0.46
340	19.90 ± 0.01	0.81 ± 0.01	0.59 ± 0.02	9.58 ± 0.27

## Conclusions

We reported a novel fabrication route through the physical vacuum deposition of PbCl_2_ layers and the following MAI vaporization-assisted perovskite growth. The optical absorption and XRD spectra verified the formation of highly crystalline and pure perovskite layers. High quality, compact, and pinhole-free perovskite layers were confirmed with the average grain size of ~ 320 nm. The regular type planar heterojunction perovskite solar cells were fabricated by employing TiO_2_ and spiro-OMeTAD as electron and hole transporting layers, respectively. The champion cell showed the best efficiency of 11.5% with a small deviation, which means the good reproducibility and uniformity of the perovskite layers produced by this vapor-processing route. As a future work, it is necessary to further develop perovskite layer quality with optimizing device structure to improve the efficiency and reduce the hysteresis behavior while maintaining the benefits of the synthetic route.

## Additional file

Additional file 1: Supplementary information. (PDF 187 kb)
