# Supplementary material for: Sequentially Vapor-Grown Hybrid Perovskite for Planar Heterojunction Solar Cells
Source: Nanoscale Res Lett. 2018 Jan 11;13:9. doi: 10.1186/s11671-017-2401-5 (PMC5764897; doi:10.1186/s11671-017-2401-5)
Supplement: Additional file 1: — Supplementary information. (PDF 187 kb) [file 11671_2017_2401_MOESM1_ESM.pdf]

Supplementary Information

Sequentially Vapor Grown Hybrid Perovskite for Planar Heterojunction Solar Cells

Won-Gyu Choi[a], Dong-Won Kang[b], Sungjae Na[a], Chan-Gyu Park[a],

Fatma Pinar Gokdemir[a,c]* and Taeho Moon[a]*

[a]Department of Materials Science and Engineering, Dankook University, Cheonan 31116, Korea

[b]Department of Solar and Energy Engineering, Cheongju University, Cheongju28503, Korea

[c]Department of Physics, Yildiz Technical University, Istanbul 34210, Turkey

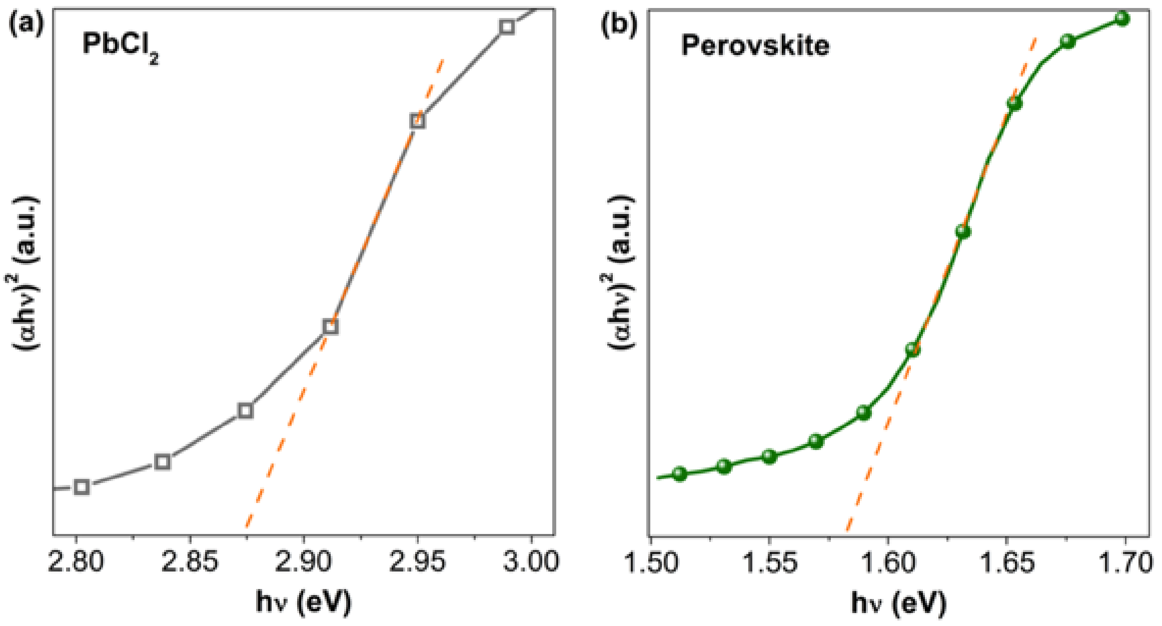

FIG. S1. Tauc plots of the absorption spectra, with linear fits near band edges. (a) $PbCl_2$. (b) Perovskite.

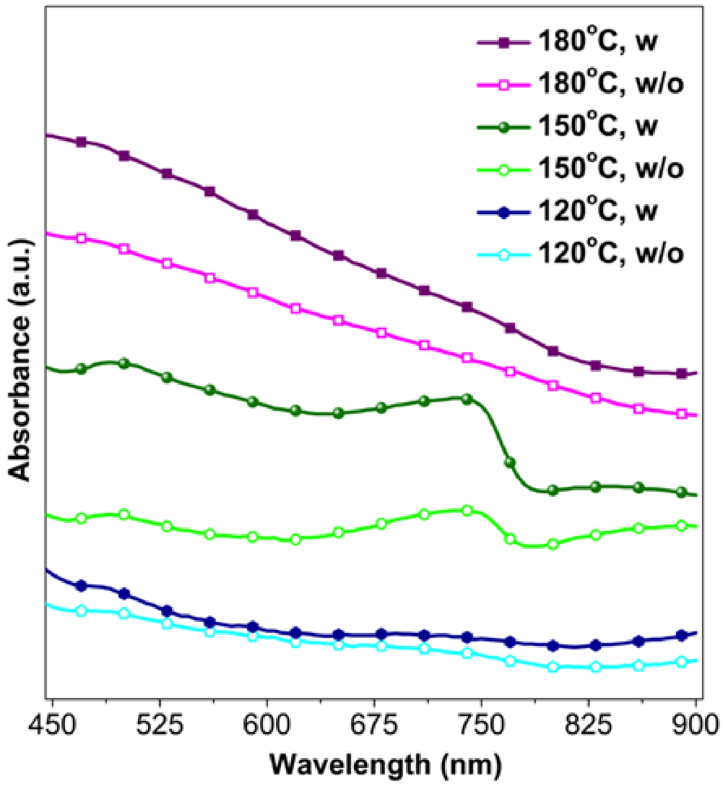

FIG. S2. UV-Vis absorption spectra of the perovskite layers, according to MAI-processing temperature and post-annealing execution (w: annealed at 100℃ for 1 h, w/o: without annealing).

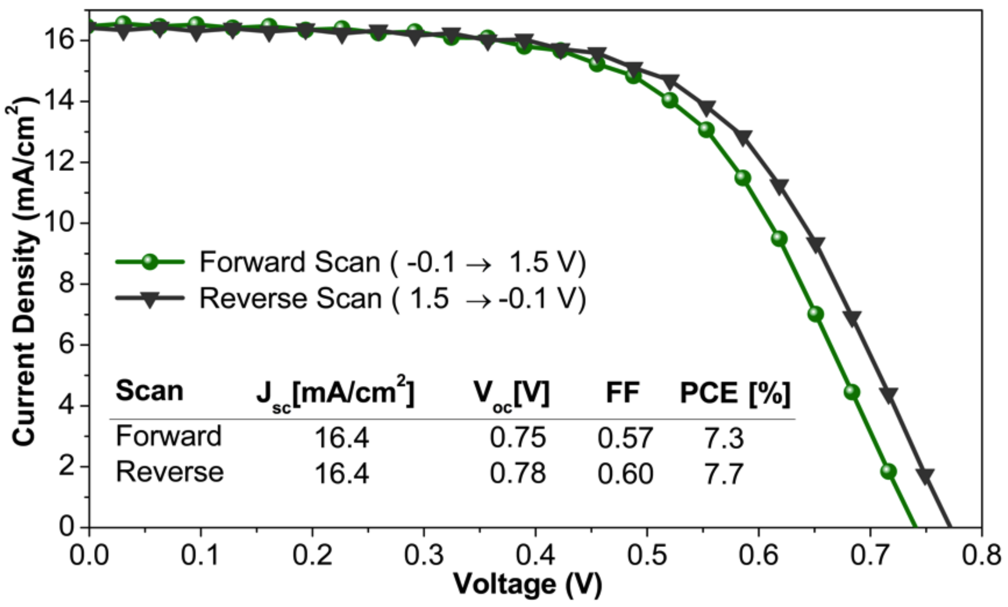

FIG. S3. Forward and reverse *J*-*V* curves at a 300 mV/s scan rate. Perovskite thickness: 220 nm.
